# From Copper Tolerance to Resistance in *Pseudomonas aeruginosa* towards Patho-Adaptation and Hospital Success

**DOI:** 10.3390/genes13020301

**Published:** 2022-02-04

**Authors:** Maxine Virieux-Petit, Florence Hammer-Dedet, Fabien Aujoulat, Estelle Jumas-Bilak, Sara Romano-Bertrand

**Affiliations:** 1 HydroSciences Montpellier, IRD, CNRS, Montpellier University, 34093 Montpellier, France; maxine.virieuxpetit@orange.fr (M.V.-P.); florencehammer@hotmail.fr (F.H.-D.); fabien.aujoulat@umontpellier.fr (F.A.); estelle.bilak@umontpellier.fr (E.J.-B.); 2 Hospital Hygiene and Infection Control Team, University Hospital of Montpellier, 34093 Montpellier, France; 3 UMR 5151 HSM, Equipe Pathogènes Hydriques Santé et Environnements, U.F.R. des Sciences Pharmaceutiques et Biologiques, Université Montpellier, 15, Avenue Charles Flahault, BP 14491, CEDEX 5, 34093 Montpellier, France

**Keywords:** *Pseudomonas aeruginosa*, copper homeostasis, copper tolerance, co-selection, environmental persistence, environmental success, healthcare-associated infection

## Abstract

The hospital environment constitutes a reservoir of opportunistic pathogens responsible for healthcare-associated infections (HCAI) such as *Pseudomonas aeruginosa* (*Pa*). *Pa* persistence within technological niches, the increasing emergence of epidemic high-risk clones in HCAI, the epidemiological link between plumbing strains and clinical strains, make it a major nosocomial pathogen. Therefore, understanding the mechanisms of *Pa* adaptation to hospital water systems would be useful in preventing HCAI. This review deciphers how copper resistance contributes to *Pa* adaptation and persistence in a hospital environment, especially within copper water systems, and ultimately to its success as a causative agent of HCAI. Numerous factors are involved in copper homeostasis in *Pa*, among which active efflux conferring copper tolerance, and copper-binding proteins regulating the copper compartmentalization between periplasm and cytoplasm. The functional harmony of copper homeostasis is regulated by several transcriptional regulators. The genomic island GI-7 appeared as especially responsible for the copper resistance in *Pa*. Mechanisms of copper and antibiotic cross-resistance and co-resistance are also identified, with potential co-regulation processes between them. Finally, copper resistance of *Pa* confers selective advantages in colonizing and persisting in hospital environments but also appears as an asset at the host/pathogen interface that helps in HCAI occurrence.

## 1. Introduction

The opportunistic pathogen, *P. aeruginosa* (*Pa*) is a major causative agent of healthcare-associated infections (HCAI) [1,2]. *Pa* is characterized by a broad capacity to colonize and persist within hospital environments [3] especially in wet technological niches such as plumbing networks where it is qualified as Opportunistic Premise Plumbing Pathogen (OPPP) [4]. This adaptation to the hospital environment and success in HCAI is conferred by its ability to form biofilms, its metabolic versatility, and its strong resistance to antimicrobial agents marked by the emergence of Multi-Drug Resistant (MDR) or eXtremly-Drug Resistant (XDR) phenotypes. Thus, the water networks of healthcare institutions constitute technological niches frequently colonized by *Pa* [5,6,7] acting as a reservoir of opportunistic strains involved in HCAI and making *Pa* a major public health issue [8,9,10,11].

Technological niches within water systems are subject to daily pressures, such as continuous sub-chlorination of water, the presence of effluents rich in antibiotics and biocides, but also episodic pressures like decontamination cycles using hydrogen peroxide, sodium hypochlorite, or thermal shock. These pressures favour the constitution of a niche-specific bacterial microbiota adapted to microenvironmental constraints, whose involvement in HCAI has been widely demonstrated [12]. The involvement of *Pa* in HCAI and its evolving resistance have led to the emergence of MDR or XDR clinical isolates, generating complex clinical situations and difficult therapeutic management. The widespread of drug-resistant *Pa* results from the combination of multiple resistance mechanisms:

- Strong intrinsic resistance to biocides due to a lack of membrane permeability.
- Consistent or inducible expression of efflux pumps, such as MexAB-OprM and MexXY, competent for the efflux of β-lactams, fluoroquinolones, and aminoglycosides [13,14].
- The acquisition by horizontal gene transfer of genes encoding carbapenemases, which are B-lactamases that hydrolyse the majority of β-lactam antibiotics, including carbapenems (last resort antibiotics), responsible for a high level of acquired resistance and therapeutical issues [15].
- Its genomic plasticity [16] combined with the selection of adaptative mutations improving *Pa* fitness and promoting development and persistence in hospital environments [17].

An increasing number of reports highlights the significant positive correlation between MDR-*Pa* phenotype isolates and virulence factors [18]. Indeed, despite a non-clonal epidemic population structure, numerous studies have demonstrated the global dissemination and frequent involvement of multi-antibiotic resistant clones of *Pa*, called epidemic high risk (EHR) clones, in the occurrence of epidemics especially within hospitals [13,19]. The classification of *Pa* isolates, as EHR or non-EHR, is based on two essential characteristics:

- The grouping of EHR isolates within a limited number of genotypes such as ST253, ST308, ST395, ST235, ST175, ST111, and ST244 [20].
- The over-representation of EHR isolates among the MDR profiles.

However, it is interesting to keep in mind that some EHRs, such as ST308 or ST395 isolates, for example, can respond to both wild-type and MDR profiles [20]. This suggests that an essential characteristic of EHR clones, beyond their major capacity to resist antibiotics, would be the ability to acquire antimicrobial resistance determinants in case of high pressure or virulence traits facilitating their survival, establishment, and ultimately their dissemination in the hospital environment. These adaptive capacities can be cumulative and are indicative of the greater intrinsic capacity of EHRs to acquire exogenous genetic material involved in adaptation to environmental constraints [13].

The extensive use of antibiotics in recent decades and the simultaneous rise of antibiotic resistance mechanisms have led to renewed interest in the antimicrobial properties of copper [21]. Two main mechanisms explain the kinetics of bacterial cell inactivation within copper plumbing network:

- The solubilisation of copper ions, secondary to the leaching phenomenon, under the dependence of many physico-chemical parameters such as pH and dissolved oxygen concentration in water [22].
- The direct interaction of micro-organisms with the metal surface, known as “contact killing” [23].

The use of metallic copper as a coating agent for plumbing systems, healthcare surfaces, and medical devices, seems to be an interesting alternative to combat bacterial overgrowth in the healthcare environment. It may reduce the use of biocides or antibiotics in hospitals and ultimately decrease the environmental pressure they exert on the hospital microbial ecology [24,25]. However, copper itself is a source of selection pressure favouring the induction of metal resistance mechanisms in bacteria and by extension their long-term survival despite the presence of a copper surface.

For example, it has been shown in *Pa* and *Escherichia coli* that pre-exposure of strains to copper improves their survival on copper surfaces by enhancing their copper resistance capacity [26,27]. Similarly, Maertens et al. [28] demonstrated that pre-induced *Cupriavidus metallidurans* (*C. metallidurans*) cells are better equipped to cope with the presence of a Cu copper plate. Indeed, the exposure of *C. metallidurans* to high copper concentration results in the generation of a new cell state in part of the population, called the viable but non-culturable state (VBNC). This VBNC state would notably allow for increased resistance to many environmental stresses, such as antibiotics [29], oxidative stress, and metals like copper [30]. As the establishment of copper resistance mechanisms in *C. metallidurans* is slow and gradual, the VBNC state appears to be a transient survival strategy. Indeed, Martens was able to demonstrate that the induction of copper resistance mechanisms plays an important role in allowing a return to the previous state, i.e., restoration of viable cells with the recovery of the ability to grow without the addition of a copper chelating agent. The cellular mechanisms of copper tolerance and resistance contributing to the survival of *C. metallidurans* in copper-rich environments are gradually being elucidated. Nevertheless, this bacterial model is not comparable to the bacteria encountered in the hospital ecosystem. Thus, it seems important to gain a better understanding of these mechanisms, particularly in *Pa*, and to identify what impact the selection pressure exerted by antimicrobial coatings and copper water systems may have on the epidemiology of bacterial resistance in health care facilities.

Petitjean et al. [31] recently described an outbreak of *Pa* ST395, labelled as an EHR, during which plumbing systems were incriminated and the systematic copper resistance of ST395 isolates would partly explain their success in hospital outbreaks. Thus, *Pa* appears to be increasingly equipped to cope with environmental and clinical stresses. However, the role of copper resistance in *Pa* colonising healthcare plumbing systems and its involvement in HCAIs remains poorly described. It seems essential to better understand how copper resistance contributes to the adaptation and persistence of *Pa* in copper water systems. To this end, this review brings together scientific knowledge on copper (Cu) homeostasis in *Pa*, the cytosolic and periplasmic organisation of copper management in *Pa* to demonstrate the central role of copper tolerance and resistance in its survival in plumbing systems, its involvement in the co-selection of metal and antibiotic resistances and its success as a causative agent of HCAIs.

## 2. Copper Is an Ambivalent Element, Both Essential and Toxic for Bacteria

Within the bacterial kingdom, copper is an essential trace element used as a cofactor in various proteins (mainly enzymes such as redox enzymes) [32] because of its redox potential. It enables many fundamental metabolic functions in cells such as denitrification and electron transport for oxidative respiration [33]. Reduced and oxidised forms of copper have an affinity for multiple amino acids (mainly cysteine, methionine, and histidine). The diversity of physico-chemical properties offered by these amino acids’ influences copper access to proteins and allows the regulation of protein-Cu contacts [34]. Due to its oxidative power, intracellular copper in its “free” soluble form is highly reactive and toxic. Free copper catalyses the formation of oxygen free radicals, like H_2_O_2_, responsible for DNA/RNA damage and lipid peroxidation. Furthermore, soluble copper can disrupt the binding of iron or sulphur to their respective enzymes resulting in poor protein metallisation and ultimately inactivation [35,36]. Thus, bacteria have acquired useful attributes to regulate intracellular copper concentration and minimise its toxicity. Bacterial cells have adapted and become competent to acquire, transport, sequester, and export copper to maintain a homeostatic balance that allows biochemical processes essential for life to occur while preventing the accumulation of toxic levels.

Over the last two decades, the understanding of bacterial copper homeostasis and the resulting mechanisms of toxicity have progressed significantly. Numerous studies on copper tolerance [37,38] have concluded that the mechanisms governing copper balance and homeostasis are involved in tolerance mechanisms when the bacterial cell is exposed to copper. In all bacteria, copper homeostasis involves at least three types of proteins:

- A copper exporting ATPase that pumps copper through the cytoplasmic membrane;
- A copper chaperone protein, which sequesters cytoplasmic copper and regulates its transport, especially by allowing its delivery to the efflux pumps;
- A copper-sensitive metal detector that regulates the level of expression of above ATPase and chaperon protein involved in copper metabolism.

Gram-negative bacteria have additional components due to their outer membrane, such as specific copper export systems to overcome this additional barrier, periplasmic multicopper oxidases, and periplasmic copper chaperone proteins [36,38,39].

To date, there is no clear scientific consensus on the mechanisms involved in copper tolerance on the one hand and copper resistance on the other. Both terms are commonly used to describe bacterial strains whose survival is facilitated in copper-rich media or on copper-coated surfaces. The lack of a boundary between tolerance and resistance to copper is partly explained by the richness and multiplicity of mechanisms involved. In the remainder of this review, we have chosen to distinguish between tolerance and resistance to copper despite the persistent ambiguity in the scientific community. The term “tolerance” will refer to the use of mechanisms governing copper homeostasis in order to respond to variation in copper concentration in the environment of the bacteria. The term “resistance” will refer to the mechanisms behind the selective survival of certain bacteria in a toxic external environment.

According to Elguindi et al. [40], the genes involved in copper resistance influence survival on copper alloys. By extension, it can be hypothesised that these genes also facilitate the adaptation and survival of *Pa* in copper-rich environments such as copper water systems.

## 3. Copper Tolerance in *P. aeruginosa*

### 3.1. Active Efflux: An Essential Aspect of Copper Tolerance

**Figure 1:**
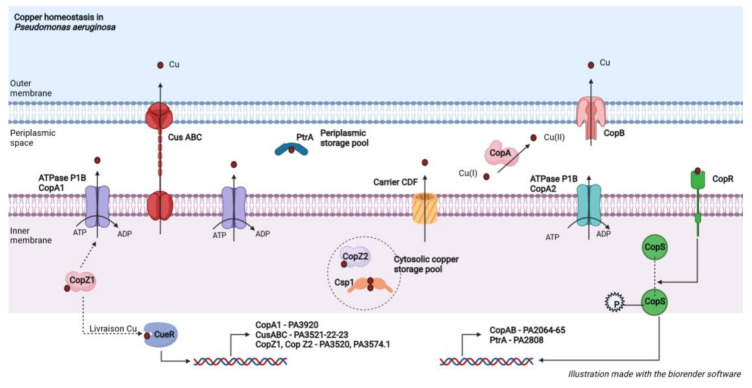
Proteins involved in the copper homeostasis of *P. aeruginosa*. The proteins are depicted in a schematic representation.

Within *Pa*, the efflux of copper is ensured by various categories of exporting proteins, namely P1B-type ATPases, the ABC family of transporters, and the cation diffusion facilitator carriers (CDFs) (Figure 1).

The P1B ATPases are the most abundant copper exporters. *Pa* has two structurally similar Cu(+)-ATPases, CopA1, and CopA2, for the active efflux of copper through the inner membrane by using the hydrolysis of ATP as an energy source (Figure 1) (Table 1). They display different functional roles in accordance with their intrinsic kinetic characteristics regarding copper. Indeed, CopA1 has a lower affinity for Cu associated with a high transfer rate. Its expression is increased when the cell is exposed to high copper concentrations [41] consolidating its involvement in the regulation of Cu cytoplasmic concentration. In addition, a mutation in the PA3920 gene, encoding the CopA1 protein (formerly known as the *cueA* gene), increases *Pa* sensitivity to copper due to Cu cytoplasmic accumulation [42,43]. Unlike CopA1, CopA2 has a greater affinity for Cu and a low turnover rate. Furthermore, CopA2 is co-expressed with cytochrome c oxidase subunits, and a reduced oxidase activity is observed in a CopA2 mutant, reinforcing the hypothesis that CopA2 is more involved in the metalation of metalloproteins such as cytochrome c oxidase. Thus, beyond their role in reducing cytoplasmic copper concentrations, P1B-type ATPases may also serve as a delivery mechanism from Cu to periplasmic cupro-proteins such as CopA or as a loading mechanism from Cu to metalloenzymes such as cytochrome C oxidase [42] (Figure 1). The role of cupro-proteins is described later.

Concerning ABC transporters, the CusABC complex is a large copper exporter, organising itself into three subunits, present in the majority of γ-proteobacteria [44]. Studies on *E. coli* show that the CusABC complex mediates copper export through the inner and outer membranes and is required for tolerating moderate to high copper concentrations [45,46].

A “CusABC analogue” system, which is an RND-type multi-drug efflux pump, is found in *Pa,* probably encoded by the *mexPQ-opmE* operon [47]. The CusABC complex is composed of proton-substrate support (CusA) located in the inner membrane and a pore found in the outer membrane (CusC), both linked by a binding protein (CusB) in the periplasm (Figure 1) (Table 1). Therefore, the main function of the CusABC complex may be the direct export of Cu from the periplasm to the extracellular medium [45]. For example, in *E. coli*, substitutions of methionine residues in the periplasmic part of the CusA protein result in a loss of copper tolerance, demonstrating the functional importance of CusA [46]. However, the presence of the periplasmic proteins CusB and CusC is essential to confer complete copper tolerance [46]. The system was initially described in *E. coli* where the chaperone protein CusF enables delivery of cytoplasmic Cu to the CusABC complex [37]. However, *Pa* does not possess CusF, hence cytoplasmic Cu is probably the direct substrate of the CusABC complex in *Pa*.

Additional tolerance may be conferred by plasmid-encoded resistance determinants from bacteria isolated from environments with very high copper concentrations. In *Pseudomonas syringae*, isolated from tomato plants treated with copper-based fungicides, a copABCD system, encoding an ABC transporter pump and carried by the plasmid pPT23D, has been described [48,49]. The cop systems consist of four structural proteins, all having the same purpose, i.e., to allow the expulsion of copper from the cell:

- CopA is a soluble periplasmic protein, exhibiting strong oxidase homology and functioning similarly to the multi-copper CueO oxidase characterized in *E. coli* [50]. In a simplified manner, multicopper oxidases perform the oxidation of Cu-(I) copper to produce Cu-(II) that is less toxic to the cell. In *E. coli*, it is recognized that the multi-copper CueO oxidase also oxidizes an enterobactin precursor [51]. Enterobactin is an iron-trapping siderophore. This siderophore has been shown to increase the sensitivity of *E. coli* to copper by reducing copper [51]. Thus, oxidation of the enterobactin precursor by CueO may be an additional mechanism to prevent the generation of toxic Cu(I) [42]. By extension, from the analogy of the CopA protein from *Pa* to *E. coli* CueO, it can be hypothesized that CopA may be involved in copper tolerance by this other mechanism.
- CopB is located on the outer membrane and is involved in copper translocation.
- CopC is a copper-binding protein located in the periplasm, capable of delivering copper to CopD, whose role is not clearly described.

Similarly, a PcoAB system exists in *Pa*, encoded by the PA2065 and PA2064 genes, that appears to perform the same functions as copABCD system described in *P. syringae* (Table 1). The *Pa* genome lacks homologues for the CopCD-encoding genes present in *P. syringae* [52].

CDF carriers, such as the transporter encoded by the PA3097 gene, also contribute to metal efflux across the inner membrane [43,53,54,55] (Table 1). Moore et al. [50] reported that a member of the *Bacillus subtilis* CDF family conferred protection against Cu. The PA01 genome has two other genes encoding CDF-type transporters, namely the PA1297 and PA3693 genes (Figure 1).

### 3.2. Copper-Binding Proteins: An Essential Element for Efficient Tolerance

Among the copper-binding proteins, there are:

- Copper transport proteins, known as chaperone proteins, ensure the cytoplasmic and periplasmic circulation of copper. *Pa* has two homologous chaperone proteins CopZ1 and CopZ2 that carry out the cytoplasmic transport of Cu to the efflux ATPases (Figure 1) (Table 1). These proteins bind copper and allow coordinated and specific delivery. Indeed, there are P1B type chaperone/ATPase pairs. Thus, CopZ1 interacts specifically with the cytoplasmic amino-terminal domain of CopA1 by electrostatic interaction [56]. Despite their strong structural similarity, CopZ1 and CopZ2 appear to have quite different functional roles. According to Lorena Novoa-Aponte et al. [57], CopZ1 is capable of metallizing CueR, a key transcriptional regulator of copper metabolism. This results in the up-regulation of genes encoding copper-exporting proteins involved in metal tolerance. Under basal conditions, CopZ1 binds copper with a higher affinity than CopZ2. In the absence of stress, CopZ2 is poorly metallized. Conversely, metallic stress significantly induces CopZ2 production suggesting that CopZ2 is a copper selective, inducible, and fast responding storage protein.
- Storage proteins, capable of binding a significant amount of copper when the cell is exposed to stress conditions, i.e., high cytoplasmic copper concentrations. As an example, the PtrA protein, encoded by the PA2808 gene, is a periplasmic copper storage protein (Table 1). The synthesis of PtrA depends on the copper concentration (inducible when CuSO4 ≥ 2 mM) and the two-component regulatory system CopR-CopS. PtrA has been shown to participate in copper tolerance in *Pa* [58].

In *B. subtilis*, a protein named Csp3, involved in the cytoplasmic storage of copper has recently been characterized [59] (Table 1). Csp3 is a tetramer capable of binding up to 80 Cu(I) ions. An in vivo study showed that the Csp3 protein prevents toxicity caused by the presence of excess copper. Indeed, bacteria expressing Csp3 are competent in copper storage: they accumulate copper and maintain significant quantities of Cu+ ions in their cytosol. Csp3-producing bacteria, therefore, possess a key storage mechanism in addition to efflux to cope with copper toxicity. The literature review suggests that the Csp1 protein described in *Pa* is an analogue of the Csp3 protein of *B. subtilis*.

### 3.3. Compartmentalization of Copper Requirements: A Dynamic between the Cytoplasmic and Periplasmic Compartments

According to Parmar et al. [60], when *Pa* is confronted with external copper concentrations around 1mM, the periplasmic copper pool becomes larger than the cytoplasmic pool. The existence of periplasmic copper storage protecting against copper toxicity in case of dyshomeostasis in *Pa* is observed. This phenomenon is made possible by an upward regulation of the periplasmic cupro-protein pool in response to an increase in the level of cytoplasmic copper secondary to the entry of copper into the cell. However, at highly toxic external copper concentrations in the order of 4mM, the copper tolerance mechanisms are saturated, and a redistribution/transfer of intracellular copper is observed: the cytoplasmic copper pool becomes predominant.

### 3.4. Transcriptional Regulators: Orchestra Masters Guaranteeing Functional Harmony

In general, the expression of copper tolerance genes is increased under metallic stress by the action of “copper sensing” transcription factors [42]. These factors such as CueR act as a sensor of the intracellular concentration in free copper and mediate the transcription of tolerance genes in response to the excess of copper. Indeed, CueR controls a panel of five promoters of 11 genes mainly involved in copper tolerance, including genes encoding for CopA1, CopZ1, CopZ2, and the CusABC system (Table 1). The deletion of the gene PA4778 encoding for CueR is responsible for an increase in *Pa* sensitivity to Cu [61]. The activation of CueR is a function of the cytoplasmic copper concentration while its transcription is directly dependent on the quorum sensing (QS) [61]. Indeed, LasR, which is the main actor of the QS, binds to the promoter of the PA4778 gene. Thus, it seems consistent to say that the regulation of copper concentration is closely linked to the detection of QS and therefore to a high bacterial density.

The principal moderator of Cu homeostasis is a two-component regulator called CopR/CopS that controls, among other things, the expression of the periplasmic copper-binding proteins PcoAB and PtrA described above [37,62]. CopR is a periplasmic sensor inducing an upward regulation of several periplasmic redox enzymes in case of increase in periplasmic Cu concentration. This up-regulation suggests that oxidases decrease the toxicity of Cu(II) [63] but also provides molecular support for the early accumulation of periplasmic Cu during copper stress (Table 1). This type of regulatory system includes a detection protein (CopR) responsible for the phosphorylation and subsequent activation of the regulatory component (CopS) (Table 1). The latter is involved in DNA binding and transcription initiation [64]. In vitro studies have shown that a deficient mutant CopR strain is more sensitive to copper [40]. Indeed, the survival rate of mutant strains, in a medium containing increasing concentrations of Cu, decreases more rapidly than the wild profile to reach zero growth as soon as the copper concentration reaches 1mM. Furthermore, the fact that CopR is upwardly regulated in the event of excess copper reinforces the hypothesis that it is a key regulator involved in Cu tolerance in *Pa* [43].

The proximity of the genes encoding CopR/S with the PA2807 and PA2808 genes is interesting. These two genes are also up-regulated during Cu stress [43]. However, the PA2808 gene allows the production of the Cu storage protein PtrA described above and the PA2807 gene codes for a hypothetical protein of the plastocyanin/azurin family carrying a Cu binding motif (Table 1). This protein is similar to the Cot protein of *Pseudomonas fluorescens* DF57 [43]. However, Cot is a Cu-tolerant protein whose expression is increased in response to Cu [65].

### 3.5. New Candidates for Copper Tolerance

Teitzel et al. [43] showed that a group of genes belonging to the periplasmic folding system of proteins, called Dsb genes, were up-regulated in a population of *Pa* described as adapted to Cu. These Dsb genes were not known to play a role in Cu tolerance until Hiniker A et al. [66] hypothesized their involvement in the repair of non-native disulphide bonds in periplasm in *E. coli*. Indeed, Dsb proteins possess free thiol groups capable of binding copper and thus ensure the correct folding of freshly imported proteins in the periplasm. Since then, it is demonstrated that the DsbC gene is involved in Cu tolerance in *E. coli* [66].

Furthermore, the study of the proteomic profile of PAO1 in response to copper stress revealed significant increases in the synthesis of several proteins classified as hypothetical proteins [67]. These include the protein encoded by the PA3661 gene, whose fold change is +63%. It is a small protein, whose role is currently unknown, which has a non-cytoplasmic type II signal peptide, suggesting that it is ultimately localized in the outer membrane. Due to the major induction of its synthesis in response to high Cu concentrations, a role in copper tolerance can be assumed. Similarly, the protein encoded by PA2542 undergoes a fold change of +42.6%. This large 130.5 kDa protein located in the outer membrane, seems to be homologous at its C-terminus (amino acids 900-1221) with the TamB protein produced by *E. coli*. TamB collaborates with the TamA protein to form a TAM complex that constitutes a novel protein secretion system [68]. If the resulting protein of PA2542 is actually a homologue of the TamB protein, then *Pa* would be dependent on this transport system when exposed to Cu. Therefore, possessing the genetic carriers of the TAM system conferred additional adaptive power in response to metallic stress.

## 4. From Copper Tolerance to Copper Resistance: The GI-7 Islet

Several major elements demonstrate that the proteins previously cited acting in the export of copper and more generally in its homeostasis are involved in copper tolerance. According to Elguindi, Wagner, and Rensing [40] copper homeostasis genes facilitate the survival of *Pa* in a copper-rich environment. However, altogether, they may not be sufficient to induce resistance to increased concentration of copper.

The genomic support of the GI-7 islet appears as a major factor of the resistance to copper (Table 1). This islet gathers 13 genes encoding proteins involved in copper tolerance mechanisms [31,69], among which:

- The *czcC*, *czcB*, and *czcA* genes encoding RND-type multi-drug efflux pump involved in zinc and copper tolerance [70],
- CopG, a cupro protein oxidoreductase allowing the conversion of Cu(I) and Cu(II) to minimise toxic effects and facilitate export through the Cus RND transporter efflux system [71],
- An ABC copper ATPase transporter, CopZ, CopA, and CopB, whose role has been described previously,
- Two copper-binding plastocyanins.

The GI-7 islet was first isolated from the DH01 strain belonging to the successful wide-spreading (WS) ST395 genotype [31]. The authors evaluated the survival of a panel of ST395 isolates in copper-containing media. All ST395 isolates tested have the GI-7 islet and behaved similarly, surviving after 48 h of incubation in broth containing 7.5 mM CuSO_4_. This contrasts with the differences in survival observed in the 10 non-ST395 isolates where only five isolates survived under these conditions.

Since this first description, the GI-7 islet has been identified in other WS or epidemic high-risk (EHR) genotypes: the ST308 and WS ST253. The ubiquity and success of such dominant genotypes, classified as WS or EHR, in a wide range of niches in the hospital ecosystem, such as the copper water system, is due to their important adaptive capacities, particularly in relation to their ability to acquire new resistance elements through HGT.

In order to clarify the role of the GI-7 islet with facing copper, the survival of *Pa* ST308 in a copper sulphate solution was evaluated by Jeanvoine et al. [69]. In their study, the higher concentration of Cu in water contaminated by *Pa*, was 144 µg/L, which is under the maximum threshold of 2 mg/L acceptable for human health, according to French and European standards and by the World Health Organisation (World Health Organization guidelines). Then, the authors compared the survival of a representative isolate of ST308 genotype carrying the GI-7 island in a copper sulphate solution at 150 mg/L (the relevant in vivo concentration) to the *Pa* reference strains (PA01 and PA14) and a representative strain of the EHR genotype of ST235. PA01, PA14, and *Pa* ST235 do not harbour the GI-7 islet. With an identical initial bacterial inoculum, the proportion of living bacteria after 24 h in a copper sulphate solution dosed at 150 mg/L was 10^−1^ for *Pa* ST308 versus 10^−6^ for PA01 and PA14 and 10^−5^ for *Pa* ST235 [69]. The authors concluded that the better survival of *Pa* ST308 in the presence of Cu can be attributed to the GI-7 islet since its deletion abolishes the resistance of *Pa* ST308 to copper. Indeed, *Pa* ST308 deprived of GI-7 sees its survival strongly decreased, becoming identical to that of PA01, PA14, *Pa* ST235. Consequently, the GI-7 islet is a major determinant of copper resistance and must play an important role in bacterial adaptation to water networks in copper. In hostile environments, it allows the survival of bacteria possessing it at the expense of bacteria that are lacking this islet.

Thus, the GI-7 islet offers *Pa* the capacity to adapt and colonise copper water networks in the long term, as observed for *Pa* ST395, responsible for an 11-year outbreak in the hospital of Besançon for which the water network was incriminated [31]. Consequently, the colonisation of the water network by *Pa* increases the risk of contamination of regularly used water points, constituting a proximity reservoir facilitating the transmission of *Pa* to patients.

## 5. Copper Selective Pressure and Resistances

As a saprophytic agent in the environment, *Pa* is confronted daily with metal ions such as copper. Copper is a widespread contaminant of soils and waters, but also a commonly used clinical antimicrobial agent, a key resource involved in bacterial predation by amoebae, and a central component of antibacterial responses of innate immunity in humans. The abundant sources of Cu exposure only increase the pressure it exerts on bacterial populations, leading to a natural selection of inherently copper-resistant bacteria [72,73]. Copper stress may induce a change of state in *Pa* towards VBNC state. There is also now ample evidence of a positive correlation between heavy metal resistance and antibiotic resistance, due to the selective pressure of metals in the environment. The co-selection of antibiotic and metal resistant bacteria is a major public health issue mainly because it can promote the maintenance and thus the spread of antibiotic resistance in the absence of antimicrobial pressure [74]. This co-selection is the result of three distinct phenomena: copper-antibiotic cross-resistance, copper-antibiotic co-resistance; and a co-regulation process between copper and antibiotic resistance.

### 5.1. Copper and VBNC State

Water distribution and plumbing systems are hostile environments for bacterial growth (scarcity of nutrients, presence of inhibitors such as copper and chlorine). These conditions favour the development of VBNC cells, which are not detectable via routine monitoring techniques because they cannot be cultivated on media where their development is classically observed [75].

Indeed, exposure to copper at sublethal doses, due to the oxidative stress generated, can lead to the generation of a VBNC state in *Pa* [75,76]. The transition to the VBNC state is a cell-programmed phenomenon and is not the consequence of sustained cellular damage. It is an active induction of a regulated cellular state, a secondary adaptive response to metal stress [77].

The VBNC state shares common features with the quiescent cell state and is defined by a loss of cultivability associated with the maintenance of viability characteristics (intact cell membranes and existence of metabolic activity and gene expression) [77]. It allows *Pa* to reorient its metabolism in order to repair the damage suffered and to induce copper tolerance mechanisms by the synthesis of an arsenal of efflux pumps, multi-copper oxidases, storage proteins…[78,79]. The VBNC state is the result of bacterial evolution, governed by the joint presence of *Pa* and copper, towards resistance to copper. The main danger underlying the presence of *Pa* VBNC in hospital copper water systems is the impossibility of detecting these cells by conventional culture methods used in epidemiological surveillance. Escaping the surveillance process makes *Pa* VBNC a latent threat, as the VBNC state is reversible and *Pa* retains its resuscitation potential as well as its virulence and pathogenicity traits [80,81]. Thus, this state can be considered as a step towards *Pa* patho-adaptation to the copper water system.

### 5.2. Copper and Antibiotic Cross-Resistance

**Figure 2:**
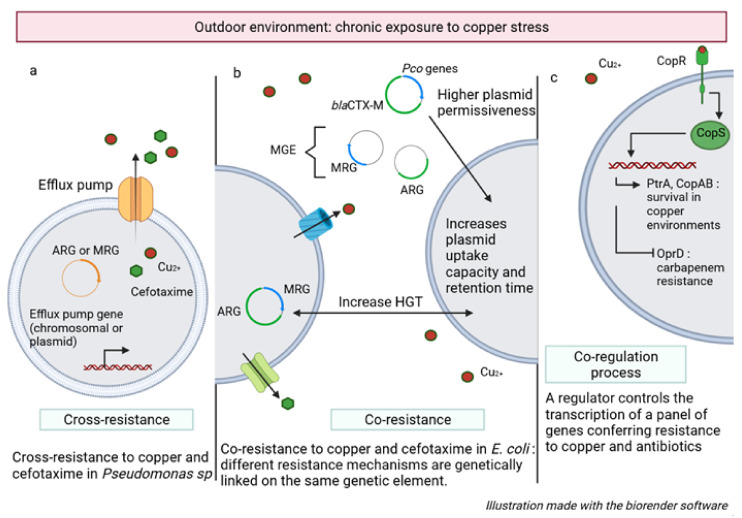
The co-selection of antibiotic and metal resistant bacteria. (**a**) cross-resistance; (**b**) co-resistance; (**c**) co-regulation process.

Due to its ability to survive and colonize water systems, especially copper, *Pa* is exposed to low concentrations of copper on a continuous and prolonged basis. This exposure is responsible for the upregulation of genes involved in active copper transport, enhancing the detoxification capacity of *Pa*’s cytoplasm and periplasm, and ultimately conferring greater copper resistance [27]. This metal resistance frequently involves an arsenal of efflux pumps that can contribute to antibiotic efflux. This phenomenon is known as cross-resistance (Figure 2a). For example, in the study conducted by Silva [82], in a river bacterial community exposed to copper concentrations between 50 and 100 µg/L (corresponding to environmentally relevant concentrations), the most represented genus showing resistance to cefotaxime was *Pseudomonas*. Teixeira [83] demonstrated that in *Pa*, the co-selection mechanism of cefotaxime and copper resistance is largely due to cross-resistance involving efflux pumps (Figure 2a). In this case, there is not necessarily a physical link between the metal and the antibiotic resistance genes. The genes are not necessarily carried by the same plasmid or clustered on the same mobile genetic element. The gene involved in resistance may be chromosomal [84]. Thus, the problem of antibiotic-metal cross-resistance is partly explained by the involvement of a large panel of genes, not specific to one type of resistance, encoding efflux pumps capable of handling a wide variety of substrates (Figure 2a).

This assertion is verified by Silva [82] who demonstrates by qPCR, that the richness of antibiotic resistance genes in the copper-exposed bacterial focus is not higher than in the unexposed control microcosm. Indeed, the absolute abundance of these genes, such as blaCTX-M and blaTEM (genes encoded for an extended-spectrum betalactamase), was below the limit of detection. He even noted a reduction in the abundance of the *tetA* gene (gene conferring resistance to tetracycline antibiotics) within the copper environment. These observations support the hypothesis that the co-selection effect is partly related to the selection of bacteria expressing intrinsic cross-resistance mechanisms (Figure 2a).

### 5.3. Copper and Antibiotic Co-Resistance

Metals can affect the distribution of antibiotic resistance genes through the selection of mobile genetic elements as shown for urban and semi-urban soils [85]. It is known that a significant proportion of antibiotic resistance genes (ARGs) are carried by mobile genetic elements (MGEs) [86]. In 2019, Zhao [85] demonstrates the existence of a significant correlation between the concentration of metals in soils and both levels of ARGs and MGEs. Thus, another correlation could explain the metal/antibiotic co-selection, distinct from the phenomenon of cross-resistance (Figure 2b). Indeed, by favouring the selection of MGEs, known to be an important source of hosts/reservoirs for ARGs, copper has an impact on the diversity, abundance, and mobility of a wide spectrum of ARGs.

The copper-induced abundance of MGEs is manifesting by an increase in ARGs abundance but also an increase in ARGs diversity [85,87]. Thus, copper exposure appears to increase the potential for horizontal transfer of ARGs [88,89]. This hypothesis is reinforced by the observation made by Cyriaque V et al. [90] that chronic metal pollution of rivers favours the transfer of exogenous plasmids to specific bacteria, previously selected by metal stress. By extension, chronic exposure to copper will result in phenotypes with intrinsically higher plasmid permissiveness. In the long term, it can be assumed that copper pressure increases plasmid uptake capacity and retention time, even for those that do not code for metal resistance (Figure 2b).

Finally, under metal stress, there is an increase in horizontal gene exchange between strains of the same species but also between species, facilitating bacterial adaptation through the acquisition of important adaptive traits such as resistance to antibiotics [91]. Copper-induced selection of MGEs can lead to the selection of MGEs carrying both copper resistance genes and ARGs. In this case, the co-selection phenomenon is explained by a physical link between the metal resistance genes and the antibiotic resistance genes. This is called co-resistance since the different resistance mechanisms are genetically linked to the same genetic element. Zhao [85] proposed it as the main co-selection mechanism for ARGs with no known role in metal resistance, i.e., ARGs that do not code for multiple efflux pumps.

A significant amount of these co-resistance plasmids is found in opportunistic pathogens. Thus, co-selection by copper, and more broadly by metals, may have important clinical implications [92] (Figure 2b).

### 5.4. Co-Regulation between Copper and Antibiotic Resistances

In addition to co- and cross-resistance, the process of co-regulation can promote the mechanism of co-selection. Co-regulation occurs when a single regulator controls a panel of resistance genes conferring resistance to various substrates (Figure 2c). Upon exposure to copper, *Pa* undergoes the first response via CueR, followed by a second response via the periplasmic CopR/S system, ultimately leading to the transcription of a gene complex that promotes the survival of *Pa* in the toxic niche (Figure 2c). The two-component system activated by high concentrations of periplasmic copper, beyond its role in copper tolerance, is responsible for an indirect down-regulation of OprD synthesis, resulting in resistance to imipenem [93].

A multitude of studies has already demonstrated the correlation between copper resistance and resistance to some antibiotics [94,95,96]. However, correlation does not imply a causative link. It is true that most studies focused on the overall changes in the abundance of ARGs, MRGs (metal resistance genes), or MGEs occurring within a bacterial community following metal exposure, without considering the parallel taxonomic changes occurring within that community. The chronic presence of an antimicrobial compound such as copper can cause a qualitative change in the community equivalent to the community-specific wealth. Indeed, several studies have demonstrated this species sorting in favour of inherently resistant populations in copper-polluted environments, without genetic changes occurring [97]. Species sorting, generated by the presence of copper, could generate a new community mostly composed of resistant strains considered pathogenic and commonly involved in infection. By extension, metal-induced selection could simply involve species that also prove to be resistant to an antibiotic. This scenario is completely different from the hypothesis that metals select for specific strains within species that have also acquired antibiotic resistance. In this second hypothesis, metal pressure would contribute to the fixation of a resistant genotype within a bacterial species independently of the antibiotic exposure pressure (Figure 2c). Conversely, in the first case, the disturbances generated by exposure to metals can be explained by variations in the composition of the bacterial community, leading to the selection of resistant bacterial species within a panel of sensitive and resistant species and not the selection of resistant sub-populations within the same species.

There are three distinct mechanisms that can explain the co-selection of metal and antimicrobial resistance:

- Cross-resistance occurs when single genes code for resistance to both antibiotics and metals (the same genetic determinant is responsible for both antibiotic and metal resistance).
- Co-resistance occurs when the genes coding for antibiotic and metal resistance are on the same mobile genetic elements (MGE like a plasmid, integron, or transposon). It is the physical link between the separate resistance genes that result in the antimicrobial and metal resistance phenotype.
- The process of co-regulation reflects the activation of a single/identical transcriptional regulator, after exposure to different stresses, resulting in the expression of a panel of genes involved in metal and antibiotic resistance.

## 6. Copper Resistance in *Pa* and Consequences in Hospitals

### 6.1. The Epidemiological Cycle of Pa in HCAIs

*Pa* is a major agent of HCAIs, especially in intensive care units or in immunocompromised patients. While the control of host-specific risk factors remains difficult, it is possible to act on the environmental risks of exposure to water or wet surfaces contaminated by *Pa* [98]. The microbial load in patient care environment plays a key role in the dispersion and transmission of HCAIs. Thus, controlling the microbial contamination threshold on surfaces close to the patient seems to be a major element to prevent the exogenous acquisition of *Pa*, which is partly avoidable.

In healthcare institutions, water networks are usually fed with drinking water, but the water used for care must meet with higher microbiological quality. Usually, drinking water and furthermore water for health care should be free of *Pa*, but the development of biofilms in the hospital plumbing system may be responsible for a random release of bacterial aggregates leading to a deterioration of the water quality [99]. Bacterial proliferation and biofilm formation are favoured by the stagnation of water or the deposits formed by corrosion [100]. Corrosion also results in the release of chemical and toxic elements such as zinc and copper, which can inactivate disinfectants, such as chlorine, and select microorganisms resistant to these metal ions [101]. Copper plumbing systems are particularly susceptible to corrosion and under the same water conditions are more frequently colonised microorganisms, especially *Legionella pneumophila* [102]. Assessing and managing the risk of waterborne infections require knowledge on water distribution facilities and the typology of the different water qualities required, but also the implementation of preventive or corrective measures to sustain the microbiologic quality of the water [6,103].

Daily flushing, temperature control in the hot water system, the fight against fouling, but mainly point-of-use treatment with 0.2 µm membrane filtration [104,105] and residual disinfection with chlorine dioxide at a concentration of 0.3 to 1 mg/L (sub-chlorination at the limit of potability) are preventive measures that have repeatedly demonstrated their effectiveness in controlling opportunistic waterborne pathogens. The implementation of point-of-use filters is a short-term solution very effective to interrupt an outbreak linked to contaminated water [106]. However, eradication of the source of water contamination remains necessary to fully protect patients. Multiple decontamination cycles (thermal or chemical shocks for example), repeated use of biocides, or increased biocides concentration are frequent strategies used for dealing with waterborne outbreaks [107]. These techniques are responsible for a drop in bacterial inoculum by killing the mesophilic flora. However, they also can be responsible for the selection of resistant bacteria such as *Pa* [108]. In case of failure of the disinfection process, further measures such as the replacement of the water network may be necessary to restore water quality. These measures are costly and can lead to the transfer of patients to another care unit or even the closure of the building.

**Figure 3:**
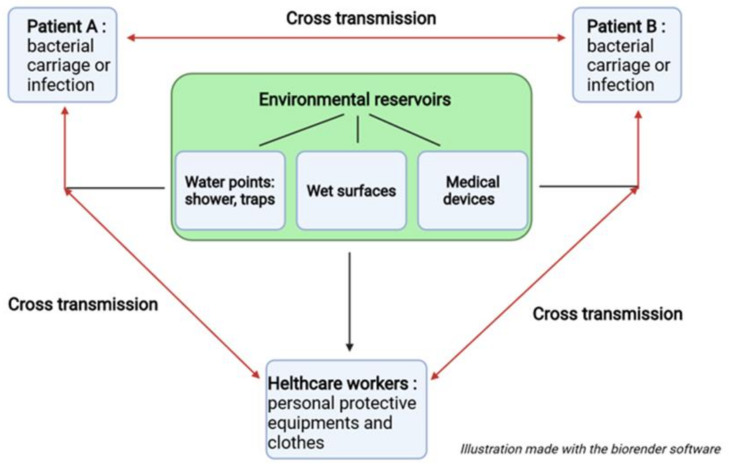
Epidemiological cycle of *P. aeruginosa* in the hospital environment.

The colonisation and persistence of *Pa* in environments, such as water systems, induce a risk of HCAI occurrence. The epidemiological cycle of *Pa* in HCAIs is complex and the hospital environment acts as a reservoir and amplifier of *Pa* [109]. The patient can become infected after exposure to contaminated water at the point of use [98,110] and then becomes a vector for the pathogen by allowing its spread in the environment (Figure 3).

Furthermore, *Pa*’s ability to cope with copper stress facilitates its survival and ultimately its perennial establishment within copper water systems [31]. Thus, copper resistance constitutes a selective advantage for the persistence of *Pa* within water systems. Similarly, its ability to form biofilms acts as an asset in colonising the hospital environment, making it more resistant to antimicrobials and allowing it to survive on many surfaces such as glass and steel, widely used for healthcare surfaces and medical device design. Prevention of HCAIs caused by *Pa* then requires controlling transmission through water and/or contaminated wet surfaces, which can be largely involved in cross-contamination [111].

This issue is even more important knowing that prolonged exposure of isolates to hospital selection pressures such as clinical effluents and chemicals promotes the emergence of multidrug-resistant isolates via co-selection of antibiotic resistance and multiple metal tolerance. This co-selection is widely documented in *Pa* [112].

### 6.2. The Use of Copper in Hospitals

Among the strategies to control surface contamination, coating surfaces with a bactericidal compound such as copper is one solution to lower the microbial load to prevent the spread of bacteria in the hospital environment. This strategy is particularly used in water supply systems constructed in copper. Several studies describe the antimicrobial and anti-biofilm properties of copper metal surfaces [113,114,115]. For example, Michael G. Schmidt, in 2012 [116], demonstrated that copper could significantly reduce the average microbial load found on copper-coated objects by 83% compared to control objects without copper. Furthermore, several studies have demonstrated anti-biofilm activity against *Pa* of copper nanoparticles (CuO NPs) with a reduction in biofilm load of more than 75% [117] or even total elimination of mature biofilm at the minimum eradication concentration of 12.5 mM [118]. Moreover, as pathogen transmission can occur during care, particularly indirectly through the professional clothing of the caregiver, the use of zinc oxide impregnated cloths is an interesting avenue to obtain a reduction in HCAIs [119].

The limitations of daily disinfection measures lie in their ability to cause a sudden but not prolonged reduction in microbial load. Thus, in combination with effective cleaning protocols, copper provides an additional strategy for the control of HCAIs due to its ability to reduce the microbial load continuously and sustainably in the healthcare environment [120] and thus provide a safer environment [121,122]. The use of copper in the healthcare environment is easy to implement as it does not require any modification of existing disinfection procedures nor generate additional annual cleaning costs. Its use as an antimicrobial agent in healthcare units is increasing, gradually replacing other materials with antibacterial properties such as silver. This trend is due to the superior bactericidal power of copper under dry conditions. Indeed, most care surfaces surrounding the patient are dry because residual moisture in the rooms does not form condensation on the surfaces. The antimicrobial superiority of copper in dry conditions is important to limit cross-contamination between a primary and secondary contaminated surface with human skin [123]. Thus, only copper-containing surfaces are truly competent to reduce HCAIs [124,125].

### 6.3. Copper Resistance: An Asset towards Bacterial Patho-Adapation

Copper, in the same manner as zinc, is an essential metal for the host’s defence. A deficiency of one of the two elements induces an increased susceptibility of the host to bacterial infections: this is the concept of nutritional immunity [126]. Copper deficiency has been shown to sensitize the host to various pathogens [127,128] while supplementation preserves against *E. coli* induced mastitis in cattle for example [129]. Indeed, a deficiency of copper leads on the one hand to neutropenia [130,131], and on the other hand neutrophils isolated from hypocupremic patients have an altered phagocytic power and a decrease in their bactericidal capacity [132]. A similar effect is observed in macrophages. The introduction of copper supplementation in hypocupremic children fully restores the phagocytic activity of polynuclear leukocytes [133].

The function of copper within the innate immune system remains to be determined more precisely, but several recent studies suggest that exposure of pathogenic bacteria to high concentrations of copper within the host is a defence mechanism of the innate immune system [39,134]. Currently, the hypothesis that copper ions are exploited by the host, especially within phagocytes, for their antibacterial effect is widely accepted [39,135]. Firstly, it has been shown that serum copper concentrations are significantly higher during an infection, reflecting the inflammatory response [136,137]. Copper accumulates at inflammatory sites [138] probably in the form of ceruloplasmin, a protein secreted by the liver during the acute phase of an inflammatory response, capable of binding six Cu ions at its active site [139]. Direct evidence that copper is recruited at infection sites has been provided by Subashchandrabose S et al. [140] by revealing a significant accumulation of Cu in the urine of patients with urinary tract infection by uropathogenic *E. coli* compared to the urine of healthy subjects. Furthermore, analysis of the microenvironment of a phagosome containing a pathogenic mycobacterium such as *Mycobacterium tuberculosis* (*M. tuberculosis*), showed an increase in copper level compared to the copper level of a phagosome containing an avirulent mycobacterium such as *Mycobacterium smegmatis* [141].

**Figure 4:**
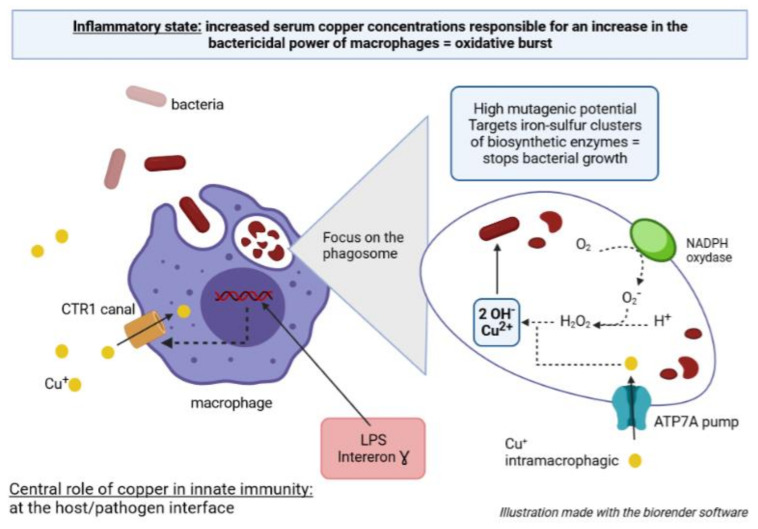
Central role of copper in innate immunity: at the host/pathogen interface.

In view of these observations, one could hypothesize that the massive mobilization of copper towards the site of infection supports the bactericidal activity of phagocytic cells, by reinforcing the “oxidative burst” produced by the macrophages. It is known that bacterial phagocytosis by macrophages leads to the formation of a phagosome that undergoes a succession of maturation phenomena, including the assembly of NADPH oxidase to the phagosome membrane. NADPH allows, under aerobic conditions, the production of superoxide ions. The superoxide ion is a highly reactive and unstable radical species that, in the presence of protons, leads to the formation of hydrogen peroxide (H_2_O_2_) [142]. Under the action of pro-inflammatory agents such as lipopolyssacharide and interferon-γ, copper importers, such as the CTR1 channel, are expressed more at the plasma membrane of macrophages [143]. Copper transport within the macrophages is therefore increased and copper is delivered to the phagosome via the ATP7A pump [143] (Figure 4). Due to its oxidative potential, copper interacts with hydrogen peroxide in a reaction like the Fenton reaction and allows the production of the hydroxyl radical which has an important antimicrobial activity:

Cu^+^ + H_2_O_2_ → Cu^2+^ + OH^−^ + HO^−^.

Classically, when this reaction is triggered by ferrous ions, the hydroxyl radicals generated have a high mutagenic and therefore lethal potential. Furthermore, beyond their mutagenic and lethal capacities, highly reactive oxygen species, generated in increased quantities in cells exposed to copper compared to cells not subjected to copper stress, are deleterious for iron-sulphur clusters [35]. These iron-sulphur clusters serve as catalysts/cofactors for key enzymes involved in amino acid biosynthesis and thus ultimately bacterial growth. In addition to its mechanism of toxicity linked to oxygen, copper is also directly toxic to these enzymes since the same decreases in activity are observed under anaerobic conditions. Indeed, copper by a competitive effect disturbs the bonds of iron-sulphur clusters by displacing the binding of iron atoms. Finally, copper, by attacking the iron-sulphur clusters, alters the reducing capacity of the bacterial cytoplasm essential to the proper functioning of the biosynthesis pathways (Figure 4).

Multiple studies suggest the involvement of copper, but also zinc, in the phagosomal killing of bacteria: this is the “brass dagger” theory detailed above. Copper homeostatic changes in the host during infection constitute a response of innate immunity to the pathogen: copper appears to play a pivotal role as a defence since a positive correlation is observed between serum copper concentrations and bactericidal power of macrophages [126,127]. Thus, the ability of pathogenic bacteria to activate several of the copper tolerance pathways could partly condition their virulence and in vivo survival in the host. On this basis, bacteria that develop copper resistance appear more enable to struggle against immune defences, and copper resistance could be considered as a bacterial patho-adaptation towards infection.

It was previously demonstrated that a bacterial strain could survive in a high-pressure copper medium if it possessed the genetic keys necessary for the development of multiple copper tolerance pathways. By extension, it can be hypothesized that these environmentally resistant strains are better able to cope with metal stress during host infection. In *Pa*, suppression of ATPase CopA1 (encoded by the *cueA gene*) decreases the survival of bacteria in the spleen of infected mice. A twenty-fold attenuation of cueA mutants is observed in the spleen of mice compared to the wild profile of the bacteria [144]. Other results confirm the pivotal role of Cu-exporting P1B ATPase in the in vivo virulence of bacteria, such as *Listeria monocytogenes* [145], *M. tuberculosis* [146,147], and *S. typhimurium* [148]. Cu(+)-ATPases are involved in the virulence of Pa: they confer tolerance to copper by combating its cytoplasmic accumulation [41]. Other results confirm the pivotal role of Cu-exporting P1B ATPase in the in vivo virulence of bacteria, such as *L. monocytogenes* [140], *M. tuberculosis* [141,142], and *S. typhimurium* [143]. Consequently, the disruption of copper tolerance pathways results in sensitization of the bacteria to the host’s innate immune system. Although the above examples suggest that pathogenic success depends on the effective removal of excess Cu from the bacterial cytoplasm, the importance of controlling Cu in the periplasmic compartment should not be overlooked. There is an increase in the expression of CopA efflux ATPase in uropathogenic *E. coli* isolated from the urine of infected mice but also an induction of the *cusABC* operon, a key component for copper detoxification of the periplasmic compartment [140].

Due to the characteristics of copper plumbing systems, and in particular their similarity to the human lung (a preferred entry point for *Pa* infections), it is reasonable to assume that water systems constitute a niche that contributes to the pathogenesis of opportunistic pathogens such as *Pa* [149].

The adaptation of a bacterium to a niche is made possible especially by the development of adaptive traits, in connection with bacterial evolution, under the influence of environmental constraints. Patho-adaptation can be defined as the use of these niche-specific adaptive traits to establish infection. Within a host, and specifically in humans, these traits become virulence traits favouring the pathogenicity of *Pa*.

The constraints on water systems favour the adaptive evolution of *Pa*, with the acquisition and development of competencies (adaptive traits), ensuring its proliferation and perennial colonisation of the pipework. When *Pa* encounters a host, it can then rely on these adaptive traits to facilitate the establishment of the infection. Indeed, exposure of bacteria to copper is responsible for an induction of copper detoxification, sequestration, and export genes, which makes these bacteria less susceptible to copper-induced destruction. Furthermore, the presence of high copper levels favours the selection of copper-resistant bacteria. Thus, the adaptation of bacteria to copper stress should improve the survival of these bacteria in macrophages, where the mechanism of bactericidal activity is based on exposure to copper overload. Similarly, water systems harbour many phagocytic protozoa. Despite the evolutionary distance from our macrophages and neutrophils, the mechanisms involved in amoebic phagocytosis are very similar to those used in innate immunity. Thus, due to exposure to eukaryotic predation, bacteria commonly found in water systems such as *Pa* are probably more resistant to the host immune response. Furthermore, the potential of copper to induce a VBNC state in *Pa* is particularly strong [75,76]. This VBNC stage may constitute an adjuvant step in the patho-adaptation process of *Pa*, allowing it time to develop the mechanisms of tolerance to copper. Finally, another patho-adaptation process underlying copper is the phenomenon of antibiotic/Cu co-selection and cross-selection, providing *Pa* with an important clinical advantage.

## 7. Summary and Conclusions

In summary, *P. aeruginosa* is a ubiquitous opportunistic pathogen capable of colonising a wide range of environments. Its environmental versatility and its patho-adaptative capacities explain its frequent involvement in hospital outbreaks. Consequently, the management of *Pa*’s infectious risk within health establishments requires the control of the environment in which it evolves. Hospital water networks constitute a preferential niche for the development of *Pa* and its eradication can sometimes prove very complex. Therefore, it seems relevant to acquire a better knowledge of the biological characteristics that can favour the establishment and propagation of *Pa* within indoor networks.

The use of copper as a material for the design of these systems and the increasing resistance of *Pa* to metals and antimicrobials requires a better understanding of the potential role of copper tolerance than resistance in *Pa* colonising plumbing systems and their involvement in nosocomial infections. As described above, *Pa* seems to naturally possess the factors key in copper homeostasis that allow it to survive in copper-containing environments. In addition, selective pressures within the hospital environment act as a driving force in generating multidrug resistance phenotypes, notably by facilitating HGT. The acquisition of new mobile genetic elements ultimately allows the adaptation of *Pa* in a polluted environment. As demonstrated by Petitjean, isolate DH01, belonging to EHR ST395, involved in a large hospital outbreak originating from the copper water system, has a network of six copper transporters acquired from non-pathogenic *Pseudomonas* sp. Thus, the presence of a pollutant such as copper increases the exchange of resistance genes between environmental and pathogenic bacteria. The increase in plasmid permissiveness concerns both plasmids carrying MRGs, ARGs, and conjugated plasmids. Copper/antimicrobial co-selection is now clearly demonstrated and could facilitate the patho-adaptation and the emergence of hospital-successful EHR genotypes. This is of particular concern given that copper is an essential part of the innate immune response during bacterial infection. Phagocytic cells exploit the antimicrobial effects of copper to fight the pathogen. However, if the pathogen can hijack the immune response, it will be more likely to colonise the host on a long-term basis and adapt easily to humans. This patho-adapation appears as an essential step towards infectious success in opportunistic pathogens such as *Pa*.

It is important to bear in mind that, beyond the use of copper as an antimicrobial agent as well as a soil and water contaminant, copper selection pressure is imposed by the innate immune system during macrophagic response as well as during phagocytosis by amoebae [150]. Indeed, amoebic predation participates in the induction of copper resistance mechanisms and ultimately in the emergence of phenotypes capable of surviving in amoebae. These intra-amoebic bacterial cells constitute a pool of *Pa* isolates not detectable by classical epidemiological surveillance techniques and potentially involved in HCAI.

## Figures and Tables

**Table 1:** Copper resistance in *P. aeruginosa* & environmental success.

Protein Family	Name of Protein	Roles
**Active efflux**		
P1B ATPases	CopA1 (PA3920)	Active efflux of copper through the inner membrane
	CopA2 (PA1549)	Copper delivery to periplasmic cuproproteins and metallation of proteins
ABC Family Carriers	CusABC “analogue system” CusA (PA3522), CusB (PA3523), CusC (PA3521)	Direct export of Cu from the periplasm to the extracellular medium
	PcoAB system CopA (PA2065), CopB (PA2064)	Periplasmic oxidation of copper and export from the periplasm to the external environment
CDF transporters	PA3097, PA129, PA3693	Metal efflux across the inner membrane
**Copper binding proteins**		Carry out the cytoplasmic transport of Cu to the efflux ATPases
Chaperone proteins	CopZ1 (PA3520)	CopZ1: metallization of CueR which triggers the first adaptive response to copper
	CopZ2 (PA3574.1)	CopZ2: Copper storage protein, inducible and rapid response to copper shock
Storage proteins	PtrA (PA2808)	Periplasmic storage of copper when the cell is exposed to copper stress conditions
	Csp1	Cytoplasmic storage of copper
**Transcriptional regulators**	CueR (PA4778)	Sensor of the cytoplasmic concentration in free copper: triggered the first adaptive response to copper. Induction of gene transcription involved in copper tolerance (CopA1, CopZ1, CopZ2 and the CusABC system).
	Two-component regulator CopR/S (PA2809, PA2810)	Sensor of the periplasmic concentration in free copper: triggered the second adaptive response to copper during prolonged exposure. Induction of PcoAB and PtrA transcription.
**Copper resistance**	GI-7 island	Major factor in the resistance to copper: islet gathers 13 genes encoding proteins involved in copper tolerance mechanisms.

## Data Availability

Not applicable.
